# Supplementary material for: Inducible Expression of Several Drosophila melanogaster Genes Encoding Juvenile Hormone Binding Proteins by a Plant Diterpene Secondary Metabolite, Methyl Lucidone
Source: Insects. 2022 Apr 29;13(5):420. doi: 10.3390/insects13050420 (PMC9144306; doi:10.3390/insects13050420)
Supplement: Supplementary file 1 [file insects-13-00420-s001.zip › insects-1677277-supplementary.pdf]

Figure S1

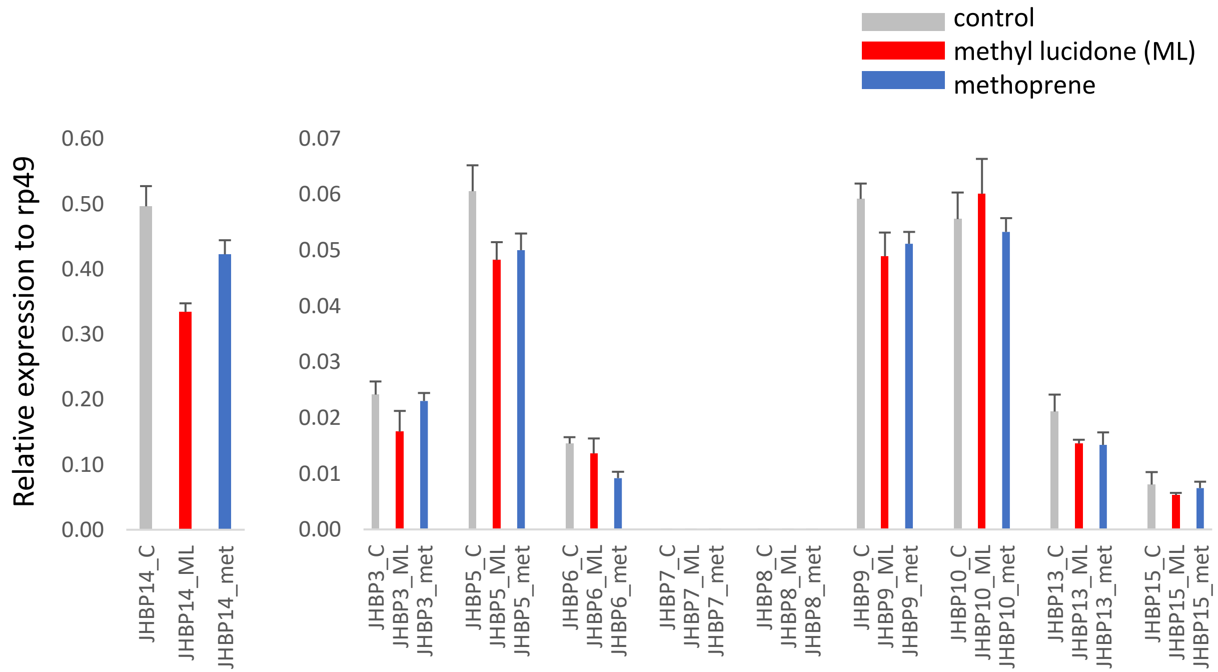

Figure S1. Ten *D. melanogaster* JHBP genes are not inducible by ML and methoprene at the 2nd instar larval stage. The gene expression was evaluated by qPCR with gene-specific primers. We show two figures with different scales of the Y-axis to clarify the expression level of each JHBP gene: the left figure depicts the relatively high abundance of JHBP14; the one on the right shows the relatively low abundance of the other nine JHBP genes. Two independent biological replicates showed that these 10 JHBP genes are not induced, and a representative result is presented. Each result shows the average value of three replicates during the RNA isolation and qPCR steps, and the error bars indicate standard deviation.

Figure S2

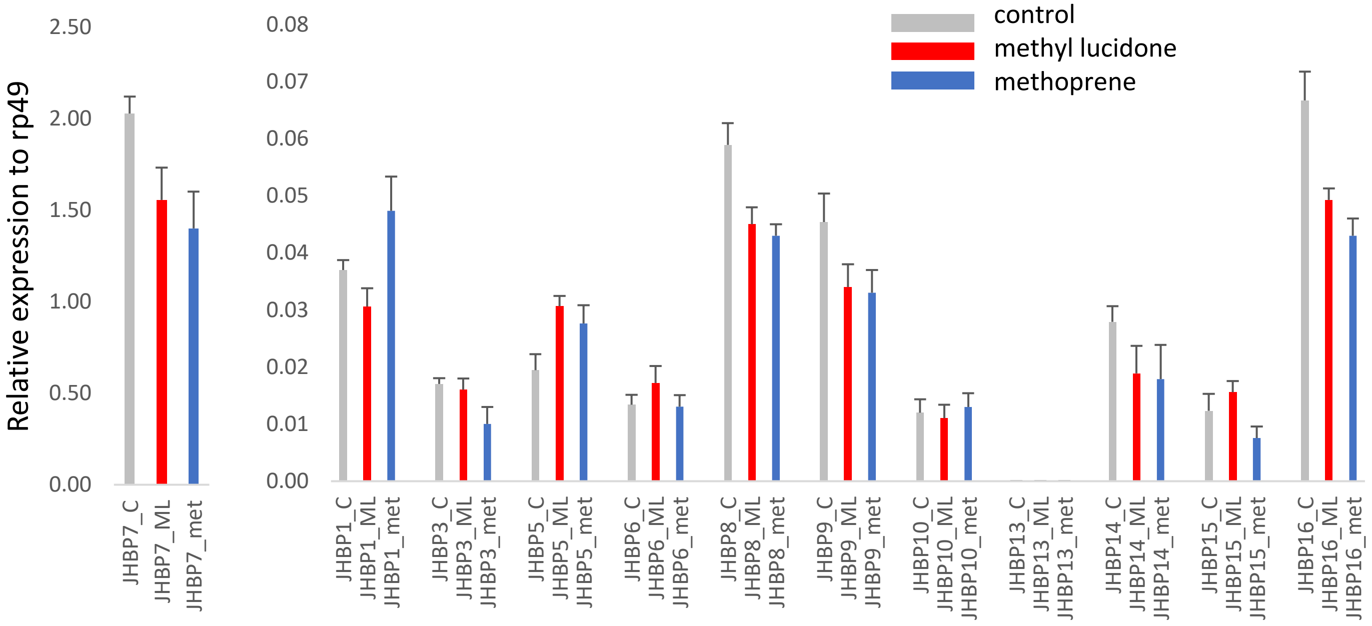

Figure S2. The expression of 12 *D. melanogaster* JHBP genes remains unchanged by ML and methoprene at the pupal stage. The gene expression was evaluated by qPCR with gene-specific primers. The figure has been split into two with different scales of the Y-axis for a clearer representation of the expression level of each JHBP gene: the left figure demonstrates the relatively high abundance of JHBP7; the one on the right shows the relatively low abundance of the remining 11 JHBP genes. Two independent biological replicates show that JHBP gene-expression levels remain unchanged, and a representative result is presented. Each result shows the average value of three replicates during the RNA isolation and qPCR steps, and the error bars indicate standard deviation.

Figure S3

JHBP2

	Adult Male		Adult Female		Larval	
Tissue	FPKM	Enrichment	FPKM	Enrichment	FPKM	Enrichment
Head	146	2.5	155	7.2		
Eye	91	1.5	58	2.7		
Brain / CNS	0.7	0.0	1.1	0.1	0.1	N.A.
Thoracicoabdominal ganglion	2.2	0.0	3.3	0.2		
Crop	19	0.3	15	0.7		
Midgut	0.3	0.0	0.1	0.0	0.1	N.A.
Hindgut	100	1.7	70	3.3	0.0	N.A.
Malpighian Tubules	0.5	0.0	1.1	0.1	0.0	N.A.
Fat body	12	0.2	12	0.6	0.1	N.A.
Salivary gland	8.3	0.1	1.7	0.1	0.1	N.A.
Heart	19	0.3	22	1.0		
Trachea					1.5	N.A.
Ovary			0.2	0.0		
Virgin Spermatheca			5.0	0.2		
Mated Spermatheca			7.0	0.3		
Testis	0.7	0.0				
Accessory glands	0.5	0.0				
Carcass	141	2.4	174	8.1	7.8	3.9
Rectal pad	15	0.3	38	1.8		
Garland cells					0.1	N.A.

Figure S3

JHBP3

Tissue	Adult Male FPKM	Adult Male Enrichment	Adult Female FPKM	Adult Female Enrichment	Larval FPKM	Larval Enrichment
Head	46	7.4	51	25		
Eye	36	5.8	36	18		
Brain / CNS	73	12	95	48	1.5	0.6
Thoracicoabdominal ganglion	1.0	0.2	2.1	1.0		
Crop	0.0	0.0	0.4	N.A.		
Midgut	0.0	0.0	0.0	N.A.	0.8	0.3
Hindgut	0.3	0.0	0.2	N.A.	1.6	0.6
Malpighian Tubules	0.0	0.0	0.0	N.A.	0.0	0.0
Fat body	0.2	0.0	0.5	N.A.	0.1	0.0
Salivary gland	0.3	0.0	0.1	N.A.	0.4	0.1
Heart	0.3	0.0	0.2	N.A.		
Trachea					63	24
Ovary			0.0	N.A.		
Virgin Spermatheca			0.5	N.A.		
Mated Spermatheca			0.5	N.A.		
Testis	0.3	0.1				
Accessory glands	0.0	0.0				
Carcass	0.8	0.1	1.4	N.A.	2.8	1.1
Rectal pad	5.3	0.9	7.3	3.7		
Garland cells					5.2	1.9

Figure S3

JHBP4

	Adult Male		Adult Female		Larval	
Tissue	FPKM	Enrichment	FPKM	Enrichment	FPKM	Enrichment
Head	0.0	N.A.	0.1	N.A.		
Eye	0.1	N.A.	0.0	N.A.		
Brain / CNS	0.0	N.A.	0.0	N.A.	0.0	N.A.
Thoracicoabdominal ganglion	0.0	N.A.	0.1	N.A.		
Crop	0.0	N.A.	0.1	N.A.		
Midgut	0.0	N.A.	0.0	N.A.	0.0	N.A.
Hindgut	0.0	N.A.	0.0	N.A.	0.0	N.A.
Malpighian Tubules	0.0	N.A.	0.0	N.A.	0.1	N.A.
Fat body	0.1	N.A.	0.0	N.A.	0.0	N.A.
Salivary gland	0.1	N.A.	0.0	N.A.	0.0	N.A.
Heart	0.0	N.A.	0.2	N.A.		
Trachea					0.1	N.A.
Ovary			0.1	N.A.		
Virgin Spermatheca			0.1	N.A.		
Mated Spermatheca			0.0	N.A.		
Testis	0.5	N.A.				
Accessory glands	0.1	N.A.				
Carcass	0.1	N.A.	0.0	N.A.	0.0	N.A.
Rectal pad	0.0	N.A.	0.0	N.A.		
Garland cells					0.0	N.A.

Figure S3

JHBP5

	Adult Male		Adult Female		Larval	
Tissue	FPKM	Enrichment	FPKM	Enrichment	FPKM	Enrichment
Head	2.4	0.0	1.8	0.1		
Eye	3.5	0.0	4.5	0.1		
Brain / CNS	0.5	0.0	0.4	0.0	1.8	N.A.
Thoracicoabdominal ganglion	1.3	0.0	1.0	0.0		
Crop	5.7	0.0	1.6	0.1		
Midgut	2.6	0.0	0.9	0.0	1.7	N.A.
Hindgut	4.5	0.0	3.8	0.1	2.3	1.2
Malpighian Tubules	11	0.1	8.8	0.3	2.5	1.2
Fat body	8.1	0.1	3.1	0.1	2.2	1.1
Salivary gland	3.4	0.0	45	1.4	1.5	N.A.
Heart	7.3	0.0	8.4	0.3		
Trachea					3.1	1.6
Ovary			29	0.9		
Virgin Spermatheca			2.5	0.1		
Mated Spermatheca			3.0	0.1		
Testis	1668	11				
Accessory glands	7.4	0.0				
Carcass	8.6	0.1	5.1	0.2	2.4	1.2
Rectal pad	2.7	0.0	3.5	0.1		
Garland cells					3.3	1.6

Figure S3

JHBP6

	Adult Male		Adult Female		Larval	
Tissue	FPKM	Enrichment	FPKM	Enrichment	FPKM	Enrichment
Head	0.2	N.A.	0.2	N.A.		
Eye	0.1	N.A.	0.0	N.A.		
Brain / CNS	0.2	N.A.	0.2	N.A.	0.0	0.0
Thoracicoabdominal ganglion	0.2	N.A.	0.2	N.A.		
Crop	0.0	N.A.	0.1	N.A.		
Midgut	11	5.4	11	5.6	7.2	3.6
Hindgut	0.4	N.A.	0.8	N.A.	0.0	0.0
Malpighian Tubules	0.2	N.A.	0.1	N.A.	0.0	0.0
Fat body	0.0	N.A.	0.0	N.A.	0.1	0.1
Salivary gland	0.1	N.A.	0.0	N.A.	0.0	0.0
Heart	0.0	N.A.	0.1	N.A.		
Trachea					0.1	0.1
Ovary			0.0	N.A.		
Virgin Spermatheca			0.0	N.A.		
Mated Spermatheca			0.0	N.A.		
Testis	1.1	N.A.				
Accessory glands	0.0	N.A.				
Carcass	0.1	N.A.	0.1	N.A.	0.0	0.0
Rectal pad	0.0	N.A.	0.0	N.A.		
Garland cells					0.5	0.2

Figure S3

JHBP7

	Adult Male		Adult Female		Larval	
Tissue	FPKM	Enrichment	FPKM	Enrichment	FPKM	Enrichment
Head	1.7	0.2	2.5	0.7		
Eye	0.3	0.0	0.5	0.1		
Brain / CNS	0.1	0.0	0.2	0.0	2.0	0.1
Thoracicoabdominal ganglion	0.9	0.1	1.1	0.3		
Crop	10	1.1	28	7.5		
Midgut	4.6	0.5	5.3	1.4	14	1.1
Hindgut	12	1.4	17	4.7	130	9.7
Malpighian Tubules	6.0	0.7	6.9	1.8	0.9	0.1
Fat body	15	1.7	7.3	2.0	3.1	0.2
Salivary gland	3.4	0.4	2.4	0.6	0.1	0.0
Heart	18	2.0	7.7	2.1		
Trachea					135	10.0
Ovary			0.9	0.2		
Virgin Spermatheca			3.2	0.9		
Mated Spermatheca			3.7	1.0		
Testis	6.9	0.8				
Accessory glands	5.6	0.6				
Carcass	8.3	0.9	6.8	1.8	21	1.5
Rectal pad	12	1.4	19	5.0		
Garland cells					19	1.4

Figure S3

JHBP8

	Adult Male		Adult Female		Larval	
Tissue	FPKM	Enrichment	FPKM	Enrichment	FPKM	Enrichment
Head	1.4	N.A.	1.1	N.A.		
Eye	3.2	1.6	2.2	1.1		
Brain / CNS	0.3	N.A.	0.3	N.A.	0.2	N.A.
Thoracicoabdominal ganglion	0.7	N.A.	0.9	N.A.		
Crop	0.9	N.A.	1.1	N.A.		
Midgut	0.5	N.A.	0.2	N.A.	0.1	N.A.
Hindgut	0.4	N.A.	0.2	N.A.	0.1	N.A.
Malpighian Tubules	0.2	N.A.	0.1	N.A.	0.1	N.A.
Fat body	0.4	N.A.	0.2	N.A.	0.0	N.A.
Salivary gland	0.6	N.A.	0.2	N.A.	0.1	N.A.
Heart	0.6	N.A.	0.3	N.A.		
Trachea					0.9	N.A.
Ovary			0.0	N.A.		
Virgin Spermatheca			0.0	N.A.		
Mated Spermatheca			0.0	N.A.		
Testis	0.1	N.A.				
Accessory glands	0.0	N.A.				
Carcass	2.2	1.1	2.3	1.1	0.1	N.A.
Rectal pad	15	7.6	0.6	N.A.		
Garland cells					0.0	N.A.

Figure S3

JHBP9

	Adult Male		Adult Female		Larval	
Tissue	FPKM	Enrichment	FPKM	Enrichment	FPKM	Enrichment
Head	132	3.6	60	7.3		
Eye	62	1.7	30	3.7		
Brain / CNS	1.5	0.0	1.1	0.1	0.0	N.A.
Thoracicoabdominal ganglion	5.6	0.2	7.9	1.0		
Crop	93	2.6	138	17		
Midgut	58	1.6	1.6	0.2	0.0	N.A.
Hindgut	73	2.0	74	9.1	30	15
Malpighian Tubules	0.2	0.0	0.9	0.1	0.1	N.A.
Fat body	0.1	0.0	0.2	0.0	0.0	N.A.
Salivary gland	23	0.6	2.1	0.3	0.0	N.A.
Heart	1.7	0.0	0.1	0.0		
Trachea					41	20
Ovary			0.1	0.0		
Virgin Spermatheca			1.1	0.1		
Mated Spermatheca			2.8	0.3		
Testis	0.2	0.0				
Accessory glands	0.1	0.0				
Carcass	13	0.4	11	1.3	0.3	N.A.
Rectal pad	519	14	229	28		
Garland cells					0.3	N.A.

Figure S3

JHBP10

	Adult Male		Adult Female		Larval	
Tissue	FPKM	Enrichment	FPKM	Enrichment	FPKM	Enrichment
Head	0.0	N.A.	0.0	N.A.		
Eye	0.0	N.A.	0.0	N.A.		
Brain / CNS	0.0	N.A.	0.0	N.A.	0.0	N.A.
Thoracicoabdominal ganglion	0.0	N.A.	0.0	N.A.		
Crop	0.0	N.A.	0.0	N.A.		
Midgut	0.0	N.A.	0.0	N.A.	0.0	N.A.
Hindgut	0.0	N.A.	0.0	N.A.	0.0	N.A.
Malpighian Tubules	0.0	N.A.	0.0	N.A.	0.0	N.A.
Fat body	0.0	N.A.	0.0	N.A.	0.0	N.A.
Salivary gland	0.0	N.A.	0.0	N.A.	0.0	N.A.
Heart	0.0	N.A.	0.1	N.A.		
Trachea					0.0	N.A.
Ovary			0.0	N.A.		
Virgin Spermatheca			0.0	N.A.		
Mated Spermatheca			0.0	N.A.		
Testis	0.0	N.A.				
Accessory glands	0.0	N.A.				
Carcass	0.0	N.A.	0.2	N.A.	0.0	N.A.
Rectal pad	0.0	N.A.	0.0	N.A.		
Garland cells					0.3	N.A.

Figure S3

JHBP11

	Adult Male		Adult Female		Larval	
Tissue	FPKM	Enrichment	FPKM	Enrichment	FPKM	Enrichment
Head	13	1.9	12	5.1		
Eye	4.3	0.6	3.6	1.6		
Brain / CNS	0.1	0.0	0.1	0.1	0.0	N.A.
Thoracicoabdominal ganglion	1.1	0.2	0.9	0.4		
Crop	0.6	0.1	0.2	0.1		
Midgut	0.1	0.0	0.0	0.0	0.0	N.A.
Hindgut	0.4	0.1	0.3	0.2	0.0	N.A.
Malpighian Tubules	0.1	0.0	0.0	0.0	0.0	N.A.
Fat body	1.1	0.2	0.4	0.2	0.0	N.A.
Salivary gland	4.9	0.7	1.7	0.7	0.0	N.A.
Heart	4.6	0.7	1.3	0.6		
Trachea					0.4	N.A.
Ovary			0.1	0.0		
Virgin Spermatheca			0.3	0.1		
Mated Spermatheca			1.5	0.6		
Testis	0.2	0.0				
Accessory glands	0.1	0.0				
Carcass	18	2.6	8.9	3.9	0.0	N.A.
Rectal pad	1.4	0.2	174	76		
Garland cells					0.0	N.A.

Figure S3

JHBP12

	Adult Male		Adult Female		Larval	
Tissue	FPKM	Enrichment	FPKM	Enrichment	FPKM	Enrichment
Head	7.2	0.2	8.8	3.0		
Eye	1.7	0.0	1.2	0.4		
Brain / CNS	0.6	0.0	1.0	0.3	0.1	N.A.
Thoracicoabdominal ganglion	0.4	0.0	0.9	0.3		
Crop	263	6.0	169	57		
Midgut	1.0	0.0	0.1	0.0	0.0	N.A.
Hindgut	8.4	0.2	11	3.6	0.6	N.A.
Malpighian Tubules	0.1	0.0	0.1	0.0	0.0	N.A.
Fat body	1.1	0.0	0.1	0.0	0.0	N.A.
Salivary gland	6.6	0.2	1.4	0.5	0.0	N.A.
Heart	2.5	0.1	0.1	0.0		
Trachea					4.9	2.5
Ovary			0.0	0.0		
Virgin Spermatheca			1.2	0.4		
Mated Spermatheca			2.1	0.7		
Testis	0.5	0.0				
Accessory glands	2.6	0.1				
Carcass	48	1.1	2.7	0.9	1.3	N.A.
Rectal pad	1143	26	693	233		
Garland cells					0.1	N.A.

Figure S3

JHBP13

	Adult Male		Adult Female		Larval	
Tissue	FPKM	Enrichment	FPKM	Enrichment	FPKM	Enrichment
Head	297	1.2	255	5.6		
Eye	265	1.1	229	5.0		
Brain / CNS	1.8	0.0	2.5	0.1	0.0	N.A.
Thoracicoabdominal ganglion	8.6	0.0	8.6	0.2		
Crop	10	0.0	9.2	0.2		
Midgut	2.1	0.0	0.4	0.0	0.0	N.A.
Hindgut	0.9	0.0	0.6	0.0	0.0	N.A.
Malpighian Tubules	0.8	0.0	0.5	0.0	0.1	N.A.
Fat body	31	0.1	9.0	0.2	0.1	N.A.
Salivary gland	26	0.1	4.9	0.1	0.0	N.A.
Heart	161	0.7	62	1.4		
Trachea					1.5	N.A.
Ovary			0.2	0.0		
Virgin Spermatheca			78	1.7		
Mated Spermatheca			72	1.6		
Testis	1.6	0.0				
Accessory glands	0.7	0.0				
Carcass	346	1.4	160	3.5	0.7	N.A.
Rectal pad	33	0.1	72	1.6		
Garland cells					0.0	N.A.

Figure S3

JHBP14

	Adult Male		Adult Female		Larval	
Tissue	FPKM	Enrichment	FPKM	Enrichment	FPKM	Enrichment
Head	214	1.8	233	6.8		
Eye	161	1.3	156	4.6		
Brain / CNS	2.8	0.0	6.8	0.2	1.7	0.3
Thoracicoabdominal ganglion	12	0.1	12	0.4		
Crop	422	3.5	1057	31		
Midgut	7.8	0.1	8.3	0.2	13	2.3
Hindgut	28	0.2	37	1.1	10	1.8
Malpighian Tubules	8.9	0.1	14	0.4	0.9	0.2
Fat body	25	0.2	17	0.5	3.4	0.6
Salivary gland	59	0.5	20	0.6	2.9	0.5
Heart	53	0.4	22	0.6		
Trachea					52	9.1
Ovary			1.7	0.0		
Virgin Spermatheca			21	0.6		
Mated Spermatheca			26	0.8		
Testis	8.9	0.1				
Accessory glands	9.2	0.1				
Carcass	134	1.1	74	2.2	17	3.0
Rectal pad	341	2.8	430	13		
Garland cells					1.9	0.3

Figure S3

JHBP15

	Adult Male		Adult Female		Larval	
Tissue	FPKM	Enrichment	FPKM	Enrichment	FPKM	Enrichment
Head	16	5.7	19	9.6		
Eye	3.8	1.4	3.7	1.8		
Brain / CNS	0.7	0.3	0.7	N.A.	0.3	N.A.
Thoracicoabdominal ganglion	0.6	0.2	0.6	N.A.		
Crop	0.8	0.3	1.3	N.A.		
Midgut	0.3	0.1	0.0	N.A.	0.2	N.A.
Hindgut	0.8	0.3	0.4	N.A.	0.0	N.A.
Malpighian Tubules	0.2	0.1	0.3	N.A.	0.1	N.A.
Fat body	0.1	0.0	1.2	N.A.	0.8	N.A.
Salivary gland	0.4	0.1	0.5	N.A.	0.5	N.A.
Heart	0.8	0.3	0.5	N.A.		
Trachea					0.3	N.A.
Ovary			0.0	N.A.		
Virgin Spermatheca			0.2	N.A.		
Mated Spermatheca			0.9	N.A.		
Testis	0.1	0.0				
Accessory glands	0.2	0.1				
Carcass	0.8	0.3	0.8	N.A.	0.1	N.A.
Rectal pad	0.1	0.0	0.4	N.A.		
Garland cells					0.8	N.A.

Figure S3

JHBP16

	Adult Male		Adult Female		Larval	
Tissue	FPKM	Enrichment	FPKM	Enrichment	FPKM	Enrichment
Head	0.4	N.A.	0.8	N.A.		
Eye	0.5	N.A.	0.6	N.A.		
Brain / CNS	0.1	N.A.	0.1	N.A.	0.3	N.A.
Thoracicoabdominal ganglion	0.2	N.A.	0.2	N.A.		
Crop	0.0	N.A.	0.1	N.A.		
Midgut	0.0	N.A.	0.0	N.A.	0.0	N.A.
Hindgut	0.1	N.A.	0.1	N.A.	0.1	N.A.
Malpighian Tubules	0.0	N.A.	0.0	N.A.	0.0	N.A.
Fat body	0.1	N.A.	0.1	N.A.	0.1	N.A.
Salivary gland	0.1	N.A.	0.1	N.A.	0.1	N.A.
Heart	0.0	N.A.	0.0	N.A.		
Trachea					1.4	N.A.
Ovary			0.1	N.A.		
Virgin Spermatheca			0.2	N.A.		
Mated Spermatheca			0.1	N.A.		
Testis	0.1	N.A.				
Accessory glands	0.1	N.A.				
Carcass	1.4	N.A.	0.3	N.A.	0.1	N.A.
Rectal pad	0.1	N.A.	0.2	N.A.		
Garland cells					0.0	N.A.
